# Contralateral parenchymal enhancement on MRI is associated with tumor proteasome pathway gene expression and overall survival of early ER+/HER2-breast cancer patients

**DOI:** 10.1016/j.breast.2021.11.002

**Published:** 2021-11-02

**Authors:** Max A.A. Ragusi, Tycho Bismeijer, Bas H.M. van der Velden, Claudette E. Loo, Sander Canisius, Jelle Wesseling, Lodewyk F.A. Wessels, Sjoerd G. Elias, Kenneth G.A. Gilhuijs

**Affiliations:** a Department of Radiology / Image Sciences Institute, University Medical Center Utrecht, Utrecht University, Heidelberglaan 100, 3584 CX Utrecht, the Netherlands; b Division of Molecular Carcinogenesis – Oncode Institute, The Netherlands Cancer Institute – Antoni van Leeuwenhoek Hospital, Plesmanlaan 121, 1066 CX Amsterdam, the Netherlands; c Department of Radiology, The Netherlands Cancer Institute – Antoni van Leeuwenhoek Hospital, Plesmanlaan 121, 1066 CX Amsterdam, the Netherlands; d Division of Molecular Pathology, The Netherlands Cancer Institute – Antoni van Leeuwenhoek Hospital, Plesmanlaan 121, 1066 CX Amsterdam, the Netherlands; e Department of Pathology, Leiden University Medical Center, Albinusdreef 2, 2333 ZA Leiden, the Netherlands; f Faculty of Electrical Engineering, Mathematics, and Computer Science, Delft University of Technology, Mekelweg 5, 2628 CD Delft, the Netherlands; g Department of Epidemiology, Julius Center for Health Sciences and Primary Care, University Medical Center Utrecht, Utrecht University, Universiteitsweg 100, 3584 CG Utrecht, the Netherlands

**Keywords:** Breast neoplasm, Magnetic resonance imaging, Gene expression, Proteasome endopeptidase complex, Parenchymal tissue

## Abstract

**Purpose:**

To assess whether contralateral parenchymal enhancement (CPE) on MRI is associated with gene expression pathways in ER+/HER2-breast cancer, and if so, whether such pathways are related to survival.

**Methods:**

Preoperative breast MRIs were analyzed of early ER+/HER2-breast cancer patients eligible for breast-conserving surgery included in a prospective observational cohort study (MARGINS). The contralateral parenchyma was segmented and CPE was calculated as the average of the top-10% delayed enhancement. Total tumor RNA sequencing was performed and gene set enrichment analysis was used to reveal gene expression pathways associated with CPE (N = 226) and related to overall survival (OS) and invasive disease-free survival (IDFS) in multivariable survival analysis. The latter was also done for the METABRIC cohort (N = 1355).

**Results:**

CPE was most strongly correlated with proteasome pathways (normalized enrichment statistic = 2.04, false discovery rate = .11). Patients with high CPE showed lower tumor proteasome gene expression. Proteasome gene expression had a hazard ratio (HR) of 1.40 (95% CI = 0.89, 2.16; P = .143) for OS in the MARGINS cohort and 1.53 (95% CI = 1.08, 2.14; P = .017) for IDFS, in METABRIC proteasome gene expression had an HR of 1.09 (95% CI = 1.01, 1.18; P = .020) for OS and 1.10 (95% CI = 1.02, 1.18; P = .012) for IDFS.

**Conclusion:**

CPE was negatively correlated with tumor proteasome gene expression in early ER+/HER2-breast cancer patients. Low tumor proteasome gene expression was associated with improved survival in the METABRIC data.

## Highlights

• Contralateral parenchymal enhancement on MRI was associated with tumor proteasome gene expression in ER+/HER2-breast cancer.
• A high contralateral parenchymal enhancement was associated with a low proteasome gene expression in the breast cancer.
• Low proteasome tumor gene expression was associated with improved survival in an independent patient cohort.

## 1 Introduction

Adjuvant systemic treatment (AST), such as endocrine, targeted, and chemotherapy, has improved the survival of breast cancer patients over the past decades [1]. Nonetheless, a substantial number of patients is overtreated with AST. Endocrine therapy can be administered to estrogen receptor-positive (ER+) breast cancer. However, besides the estrogen receptor (ER), no clinically validated options are available to support decisions to select endocrine therapy [2], despite the fact that ER + breast cancer is the most frequently occurring breast cancer subtype and endocrine therapy constitutes the largest fraction of AST administered.

A tool under investigation to personalize endocrine therapy in patients with unilateral ER + human epidermal growth factor 2-negative (HER2-) breast cancer is contralateral parenchymal enhancement (CPE) on dynamic contrast-enhanced (DCE) magnetic resonance imaging (MRI). CPE is a measure of the delayed contrast enhancement in the contralateral parenchymal breast tissue. CPE was previously associated with survival in ER+/HER2-breast cancer, but not in other breast cancer subtypes [[3], [4], [5]]. CPE was not associated with ER-percentage or with genomic ER-pathway activity of the tumor [6]. The biological mechanisms linking CPE to tumor biology, therefore, remain unknown.

The prognostic information that CPE contains, independent from routinely available clinicopathological variables (e.g. tumor size, axillary load), and genomic signatures [7], might be explained by the biological pathways expressed in the tumor. Background parenchymal enhancement (BPE; a qualitative measure of parenchymal enhancement) on MRI is a well-known independent risk factor for the development of breast cancer [[8], [9], [10]] and it may be an important indicator of the type of tumor that develops: high BPE was more strongly associated with invasive breast cancer as opposed to ductal carcinoma in-situ (DCIS) [8]. BPE was also associated with immunohistochemical subtype of the tumor, lymphovascular invasion, and tumor grade [[11], [12], [13]]. It has also been reported that breast cancer has local effects on tissue surrounding the tumor [14,15] as well as systemically on (distant) non-tumorous tissue [16,17], even before metastasis occur [18], and that these changes are associated with prognosis [[18], [19], [20]]. For example, enhancement of contralateral parenchymal tissue was associated with the presence of breast cancer (in the contralateral breast) [21], and ipsilateral parenchymal enhancement was associated with various biological pathways expressed in the tumor [19,22]. Based on these findings, we hypothesize that CPE could represent the diseased breast before tumorigenesis [4], in which case CPE could be associated with an environment that gives rise to a certain type of tumor, or that CPE is secondarily affected by tumor-induced systemic effects. In both cases CPE might be associated with biological pathways expressed in the tumor that could also affect prognosis.

The purpose of this study was to investigate whether CPE is associated with biological pathways in the tumor, and, if so, whether these CPE-associated biological pathways expressed in the tumor carry prognostic information.

## 2 Materials and methods

### 2.1 Study design

**Fig. 1:**
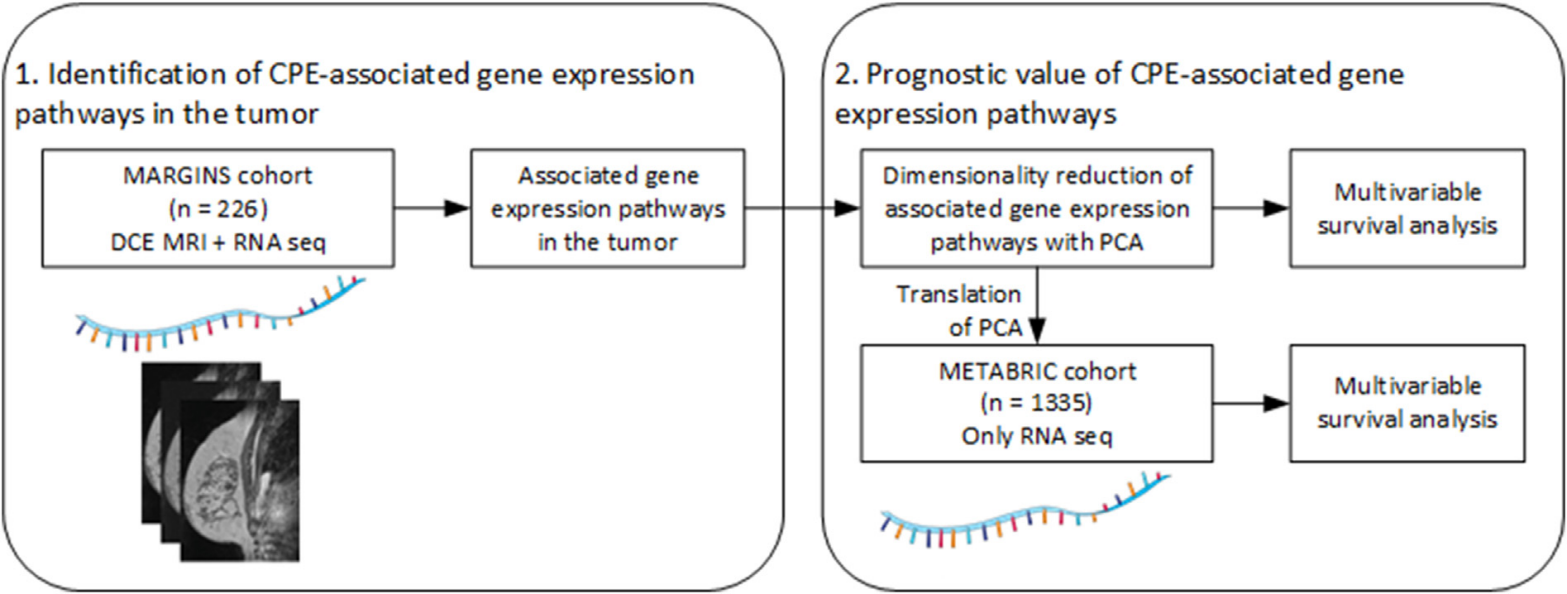
Overview of study design. This figure was created using Servier Medical Art templates, which are licensed under a Creative Commons Attribution 3.0 Unported License; https://smart.servier.com. CPE = contralateral parenchymal enhancement, MRI = magnetic resonance imaging, RNA seq = ribonucleic acid sequencing, PCA = principle component analysis, METABRIC = Molecular Taxonomy of Breast Cancer International Consortium.

To reveal biological pathways in ER+/HER2-early breast cancer that are associated with CPE and to investigate whether these CPE-associated gene expression pathways are related to survival, we performed this study in two steps. First, we identified gene expression pathways that are associated with CPE from patients included in the Multimodality Analysis and Radiologic Guidance in Breast-conserving Therapy study (MARGINS) where CPE was first described, i.e. the discovery cohort [4]. Second, the ability of these CPE-associated gene expression pathways to stratify survival was assessed, and externally verified in a publicly available dataset (Molecular Taxonomy of Breast Cancer International Consortium [METABRIC] [23], Fig. 1).

### 2.2 Patient cohort

**Fig. 2:**
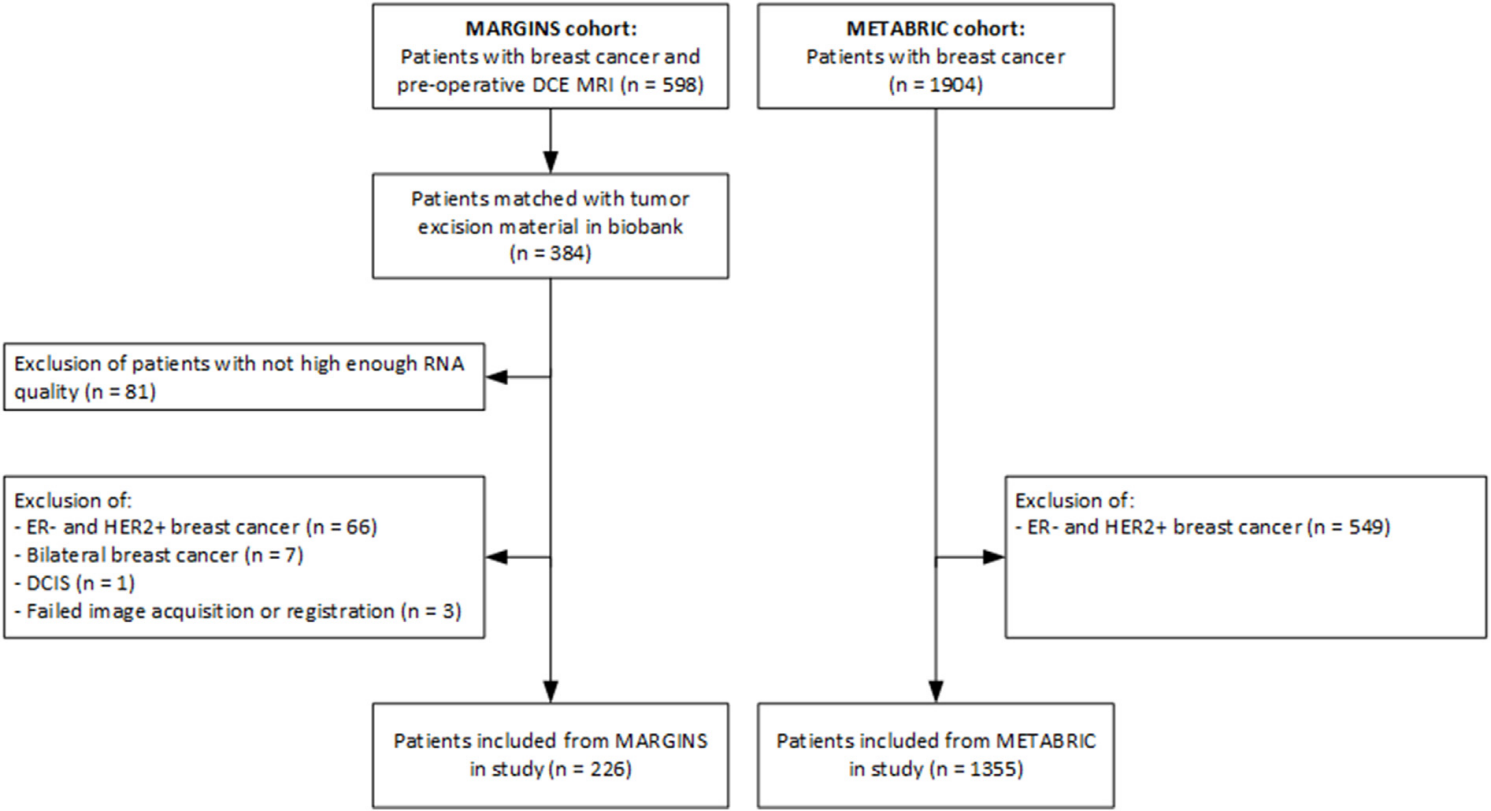
Patient inclusion chart. Low quality RNA was defined as < 30% tumor percentage or an RNA integrity number <6. MARGINS = multimodality analysis and radiologic guidance in breast-conserving therapy study, DCE = dynamic contrast-enhanced, MRI = magnetic resonance imaging, ER = estrogen receptor, HER2 = human epidermal growth factor 2, DCIS = ductal carcinoma in situ, METABRIC = molecular taxonomy of breast cancer international consortium.

This is a re-analysis of data from patients with unilateral ER+/HER2-breast cancer obtained in the MARGINS study performed between 2000 and 2008 at the Netherlands Cancer Institute. Institutional review board approval and written informed patient consent were obtained [4,24]. In MARGINS patients with proven breast cancer and eligible for breast-conserving surgery based on conventional imaging (ultrasound and/or mammography) and clinical assessment were consecutively included. These patients underwent an additional preoperative breast MRI. A total of 598 patients with breast cancer were included (Fig. 2). For 384 patients the preoperative DCE MRI could be matched to tumor material from the surgical excision in the Netherlands Cancer Institute biobank, which yielded enough high-quality RNA for sequencing in 303 patients. Patients without ER+/HER2-breast cancer (n = 67), bilateral breast cancer (n = 7), DCIS (n = 1), and with failed image acquisition or registration (n = 3) were excluded. A total of 226 patients with a preoperative DCE MRI matched with high-quality tumor RNA were included in the analysis.

### 2.3 MR imaging

The MRIs were acquired by using a 1.5-T imaging unit (Magnetom, Siemens) with a dedicated four-channel double breast array coil (Siemens). The DCE-sequence consisted of an unenhanced coronal fast low-angle shot three-dimensional T1-weighted image, followed by four consecutive contrast-enhanced series (90 s apart) after a bolus (14 mL) of a gadolinium-based contrast agent (0.1 mmol/kg, Prohance, Bracco Imaging Pharmaceutical Sterile Operations). The imaging parameters were: acquisition time 90 s, repetition time 8.1 ms, echo time 4.0 ms, a flip angle 20⁰, and voxel sizes 1.35 x 1.35 × 1.35 mm^3^ [4].

### 2.4 Contralateral parenchymal enhancement

Image processing and calculation of CPE are described elsewhere in detail [4]. Briefly, spatial variations in image intensity due to inhomogeneity of the magnetic field were corrected [25], the breast volume was segmented [26], as well as the fibroglandular tissue of the contralateral breast [27]. Post-contrast images were registered to the pre-contrast images using deformable image registration to reduce patient motion artifacts [28]. CPE is defined as the mean top-10% voxels in the contralateral fibroglandular with the highest ratio of enhancement between the early (90 s post-contrast) and late (360 s post-contrast) image: (S_late_ – S_early_)/S_early_, where S denotes signal intensity [4]. CPE is a dimensionless number.

### 2.5 METABRIC cohort

To externally validate a possible association between CPE-associated gene expression pathways and survival, gene expression data from the publicly available METABRIC cohort was used [23]. METABRIC contains clinical annotation and RNA profiles (n = 1904) derived from primary fresh frozen breast cancer specimens originating from patients from the United Kingdom and Canada (Fig. 2). We selected all patients with ER+/HER2-breast cancer resulting in a total inclusion of 1355 patients participating in METABRIC with clinical, follow-up, and tumor gene expression data.

### 2.6 Gene expression

Gene expression in the MARGINS cohort was derived from whole transcriptome RNA sequencing, as described previously [7,24]. In short, the fresh-frozen tumor samples were collected from the biobank of the Netherlands Cancer Institute. Low tumor percentage (<30%) or low RNA quality (RNA integrity number <6; Bioanalyzer 2100, Agilent) samples were excluded (Fig. 2). RNA sequencing of the samples was performed using the HiSeq 2500 (Illumina) with single-end 65 base-pair reads. RNA sequencing reads were aligned with STAR 2.5.0a to the human genome (GENCODE 23) to quantify the RNA per gene [29]. Gene expression in the METABRIC cohort was measured using microarrays. Further details about the gene expression measurements in the METABRIC cohort are described elsewhere [23].

### 2.7 Statistical analysis

#### 2.7.1 Gene expression pathway analysis

To identify gene expression pathways that are associated with CPE, we performed gene set enrichment analysis (GSEA) [30]. Firstly, CPE was regressed against all individual genes. The genes were ranked based on the strength of the association between the specific gene and CPE, quantified by the *t* statistic [31]. Based on this ranking, GSEA scored the enrichment of each gene set based on the ranking of the individual genes. To quantify the different associations between CPE and each gene set, GSEA calculated three additional scores: the normalized enrichment statistic (NES), the maximum enrichment statistic at (Max ES at), and the leading edge (LE). NES is the effect size of the gene set enrichment and can be compared between gene sets. A higher NES indicates a stronger association of CPE with that gene set. The Max ES at is the position in the ranked list at which the maximum enrichment occurred. The most relevant gene sets appear at the top or bottom of the list, i.e., have a high or low Max ES at. The leading edge is the proportion of genes in a gene set that contribute to the enrichment score. A high leading edge indicates that a large fraction of the gene set contributed to the enrichment [32]. Within the pathway analysis, differential expression on RNA sequencing data was performed using limma-voom [31]. Two gene set collections from the Molecular Signature Database (version 7.0) were used for the GSEA: c2.cgp, which contains experimentally derived gene sets (n = 3302); and c2.cp, which contains gene sets curated by domain experts (n = 2199). Together these two gene sets provide wide coverage of biological processes without being highly redundant [24]. Gene sets with a false discovery rate (FDR) < 0.25, the recommended threshold for the discovery of associated gene expression pathways [32], were considered significant and included in further analyses. Correlation of individual genes with CPE was measured with the Pearson's correlation coefficient.

#### 2.7.2 Survival analysis

To investigate whether the CPE-associated gene expression pathways were associated with survival, we fit a multivariable Cox proportional hazards model with Firth's penalized likelihood (due to the relatively low number of events) in the MARGINS cohort [33]. The endpoint was overall survival (OS) and invasive disease-free survival (IDFS) as defined by Hudis et al. [34]. The survival models were adjusted for age, tumor size, tumor grade, axillary load, and AST (yes/no). The variables axillary load and AST are highly correlated, and were added as a construct variable (i.e. the combination of both variables in a single variable, e.g. positive lymph nodes and treated with AST, negative lymph nodes and not treated with AST, etc.). We decided not to impute missing data due to the low number of cases with missing values in both the MARGINS and METABRIC cohort (2% and 5% respectively) [35]. To deal with the high dimensionality of gene expression data, a principle component analysis (PCA) was performed on the scaled gene expression data of the specific gene set, and the first principal component (PC1) was treated as the variable representative of the biological pathway in the multivariable survival model [22,36]. To validate a possible association between discovered gene expression pathways associated with CPE and survival in an external dataset, we applied the PCA from the MARGINS data to the METABRIC data, and fitted a regular multivariable Cox proportional hazards model including the PC representative of the gene expression and adjusted for age, tumor size and grade, axillary load, and AST. To translate the PCA of the MARGINS data to the METABRIC data we linearly transformed the gene expression of each gene in METABRIC to have identical mean and variance as the corresponding gene in MARGINS, because MARGINS gene expression was derived from RNA-sequencing and gene expression in METABRIC was derived from microarrays. Lastly, to increase interpretability of CPE and PC1, we standardized both variables so that a one unit increase signifies an increase of one standard deviation (SD).

Statistical analyses were performed using R version 3.6.2 (R Foundation for Statistical Computing) with the ‘limma’ (version 3.42.2) [31], ‘flexgsea’ (version 1.3), and ‘coxphf’ (version 1.13) [33] packages available in R. Descriptive statistics are reported as median with the corresponding interquartile interval (IQI), and coefficient estimates are reported with their corresponding 95% confidence interval (CI). A two-tailed p < .05 was considered to represent statistical significance.

## 3 Results

Table 1 summarizes the patient, tumor, and treatment characteristics for both the MARGINS and METABRIC cohorts. Median patient age was 59 years (IQI = 50, 64) in MARGINS and 64 years (IQI = 53, 72) in METABRIC. Patients in MARGINS underwent more breast-conserving surgery and consequently more often received radiotherapy. Additionally, the distribution of adjuvant systemic therapy (AST) differed between both cohorts: more patients were treated with only endocrine therapy in METABRIC, but less often with no AST or chemotherapy.

**Table 1:** Baseline patient, tumor, and treatment characteristics of ER+/HER2-breast cancer patients from the MARGINS and METABRIC cohorts.

	MARGINS (n = 226)	METABRIC (n = 1355)
Age (years)		
median (IQI)	59 (50, 64)	64 (53, 72)
Tumor size (mm)		
median (IQI)	19 (14, 25)	22 (17, 30)
Unknown (N)	0	12
Tumor grade		
1	80 (36%)	159 (12%)
2	112 (50%)	651 (50%)
3	31 (14%)	484 (37%)
Unknown	3	61
Axillary load		
0	142 (63%)	745 (55%)
1–3	66 (29%)	418 (31%)
4 or more	16 (7%)	192 (14%)
Unknown	2	0
Adjuvant systemic therapy		
None	122 (54%)	366 (27%)
Only endocrine therapy	49 (22%)	859 (63%)
Only chemotherapy	1 (0%)	21 (2%)
Endocrine and chemotherapy	54 (24%)	109 (8%)
CPE		
median (range)	0.438 (0.105, 0.986)	
Cause of death		
Breast-cancer	11 (61%)	388 (49%)
Non breast-cancer	7 (39%)	398 (51%)
Unknown	0	1
Breast cancer recurrence		
Yes	22 (10%)	516 (38%)
No	204 (90%)	838 (62%)
Unknown	0	1

Values are numbers of patients with percentage between parentheses, unless otherwise specified. ER = estrogen receptor, HER2 = human epidermal growth factor 2, MARGINS = multimodality analysis and radiologic guidance in breast-conserving therapy study, METABRIC = Molecular Taxonomy of Breast Cancer International Consortium, CPE = contralateral parenchymal enhancement.

### 3.1 Pathway analysis

**Fig. 3:**
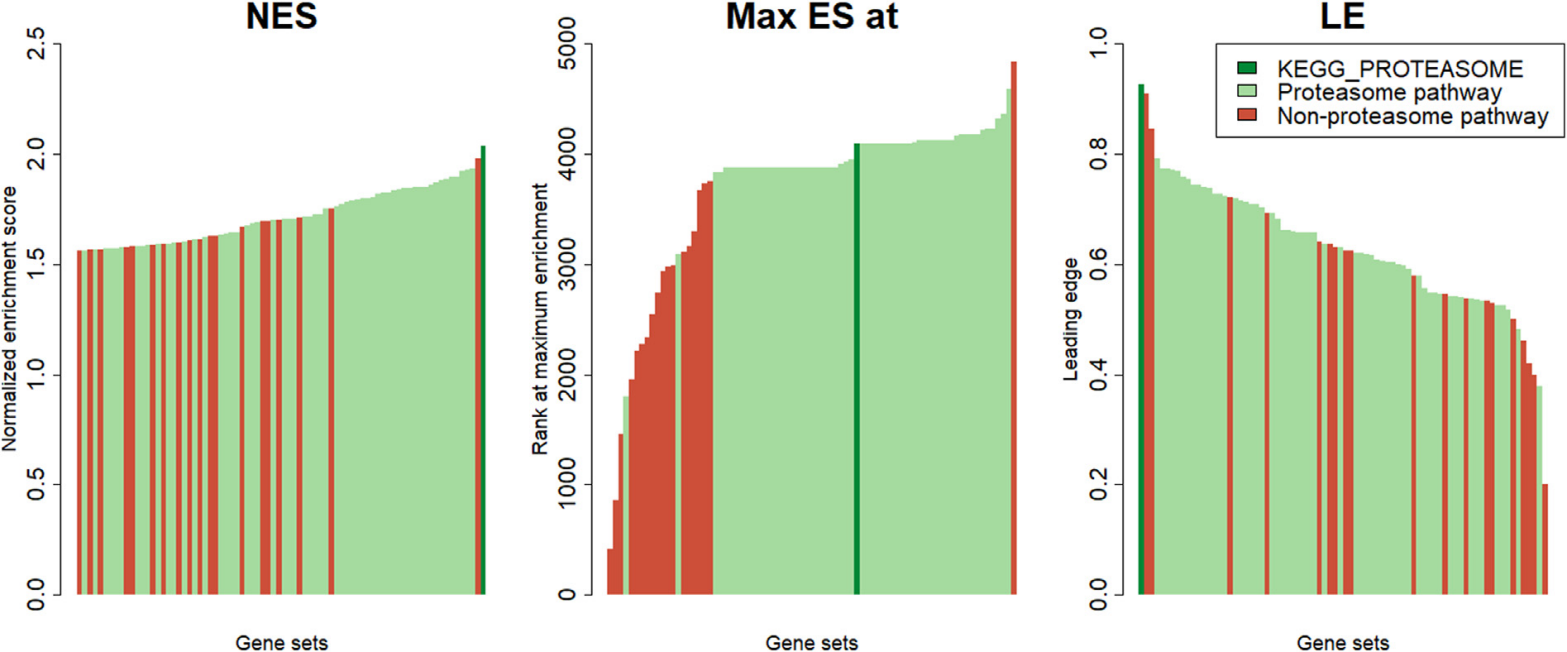
The results of the GSEA with the three association statistics of all 78 pathways with FDR < .25. CPE was most strongly associated with gene sets representing proteasome gene expression, and was most strongly associated with the KEGG_PROTEASOME pathway. NES = normalized enrichment statistic, Max ES at = maximum enrichment statistic at, LE = leading edge, GSEA = gene set enrichment analysis, FDR = false discovery rate, CPE = contralateral parenchymal enhancement.

**Fig. 4:**
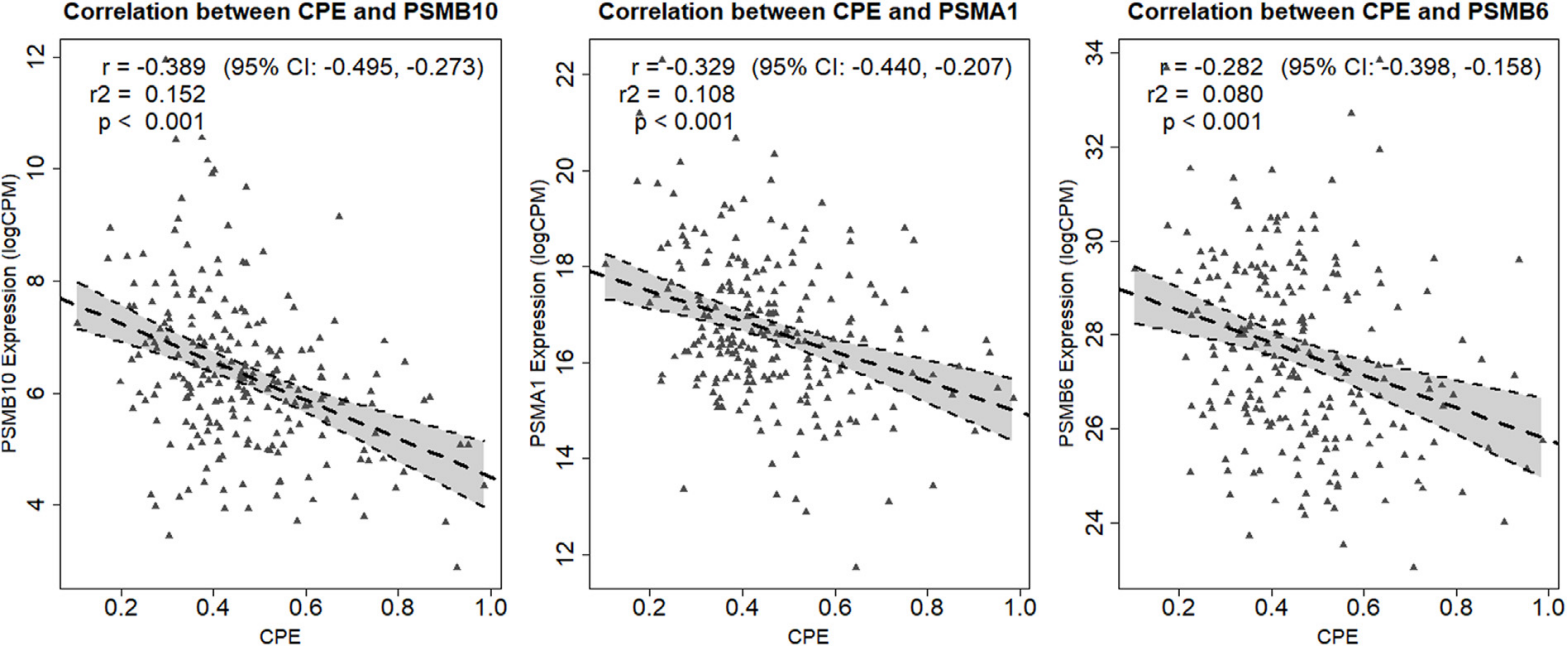
The three proteasome genes from the KEGG_PROTEASOME pathways that most strongly associate with CPE. CPE was negatively correlated with tumor proteasome gene expression, i.e. patients with a high CPE (good prognosis) had a lower proteasome gene expression on average. The regression line with 95% CI is indicated in grey. CPE = contralateral parenchymal enhancement, PSMB10 = proteasome subunit beta, PSMA1 = proteasome subunit alpha 1, PSMB5 = proteasome subunit beta 5, CI = confidence interval.

Fig. 3 summarizes the three scores (NES, Max ES at, and LE) for all 78 biological pathways associated with CPE at FDR < .25. The pathway analyses showed that CPE is strongly associated with proteasome pathways. Most notably, CPE was most strongly associated with the KEGG_PROTEASOME pathway (NES = 2.04), with high specificity (LE; 93%). Supplemental materials 1 shows an overview of all gene sets with an FDR of < 0.25 and the associated enrichment scores. Analysis of individual genes in the KEGG_PROTEASOME pathway showed that the proteasome subunit beta 10 (PSMB10) gene had the strongest correlation with CPE: 0.389 (95% CI = −0.495, −0.273; P < .001). Fig. 4 shows the three individual genes within the KEGG_PROTEASOME pathway that were most strongly correlated with CPE. Supplemental Materials 2 provides an overview for all genes in the KEGG_PROTEASOME pathway. All but one gene in the KEGG_PROTEASOME pathways were negatively correlated with CPE, i.e., patients with high CPE (favorable prognosis) had a lower tumor proteasome gene expression. Although other (non-proteasome) pathways were associated with CPE at FDR < .25, we focused on the proteasome pathways as only these pathways had both the strongest association with CPE (high NES) combined with a high proportion of genes contributing to the association with CPE (high LE; Fig. 3 and Supplemental Materials 1).

### 3.2 Principal component analysis

To investigate whether expression of the KEGG_PROTEASOME pathway is associated with survival, we first performed a PCA to condense the expression of the genes in this pathway into a single principal component to represent the KEGG_PROTEASOME pathway in the survival analysis. The first principal component explains 45% of the variance and has a correlation with CPE of −0.209 (95% CI = −0.33, −0.08, P = .002). The results of the PCA performed on the MARGINS RNA sequencing profiles were translated to the METABRIC data.

### 3.3 Survival analysis

The median follow-up was 86 months (IQI = 70, 109) with 18 OS events in MARGINS and 123 months (IQI = 73, 188) with 773 OS events in METABRIC. The median follow-up for IDFS was 84 months (IQI = 65, 107) with 30 events in MARGINS, and 108 months (IQI = 54, 170) with 804 events in METABRIC. The results of three multivariable survival models for OS in the MARGINS data adjusted for age, tumor size and grade, axillary load, and AST, are shown in Table 2: a model with only CPE, a model with only PC1 (representative of the proteasome pathway), and a model with both CPE and PC1. In the multivariable survival analysis with only CPE, CPE had a significant HR of 0.47 (95% CI = 0.23, 0.89; P = .017) per SD unit increase, i.e. patients with higher CPE have a more favorable prognosis. In the multivariable model with only PC1 (representative of tumor proteasome gene expression), PC1 had a non-significant HR of 1.40 (95% CI = 0.89, 2.16; P = .143) per SD unit increase. When modeling both CPE and PC1, the HR of CPE increased to 0.50 (95% CI = 0.24, 0.94; P = .030) and PC1 decreased to 1.26 (95% CI = 0.80, 1.94, P = .310). In the multivariable survival analysis of the METABRIC cohort, PC1 was significantly associated with survival with a HR of 1.09 (95% CI = 1.01, 1.18; P = .020). Table 3 shows the HR estimates of CPE and PC1 for IDFS, adjusted for age, tumor size and grade, axillary load, and AST. The associations between CPE and PC1 and IDFS were comparable to the associations found for OS: CPE had a HR of 0.69 (95% CI = 0.42, 1.06; P = .097) and PC1 had a HR of 1.53 (95% CI = 1.08, 2.41; P = .017). PC1 was significantly associated with IDFS with a HR of 1.10 (95% CI = 1.02, 1.18, P = .012) in the METABRIC cohort.

**Table 2:** Multivariable HR Estimates for OS for CPE and PC1 (representative of proteasome gene expression) in three models of the MARGINS cohort with only CPE, only PC1, and both CPE and PC1, and in one model of the METABRIC cohort with only PC1.

	MARGINS (n = 221)						METABRIC (n = 1283)	
Variable	Model with CPE		Model with PC1		Model with CPE and PC1		Model with PC1	
	HR (95% CI)	P	HR (95% CI)	P	HR (95% CI)	P	HR (95% CI)	P
Age (years)	1.08 (1.01, 1.14)	.015	1.09 (1.03, 1.15)	.001	1.08 (1.02, 1.14)	.012	1.05 (1.04, 1.06)	<.001
Tumor size (mm)	1.02 (0.98, 1.05)	.245	1.03 (0.99, 1.06)	.108	1.02 (0.99, 1.05)	.184	1.01 (1.01, 1.02)	<.001
Tumor grade 1	Ref		Ref		Ref		Ref	
Tumor grade 2	1.06 (0.29, 4.14)	.934	1.24 (0.34, 4.97)	.744	1.13 (0.30, 4.50)	.858	1.19 (0.91, 1.53)	.187
Tumor grade 3	1.70 (0.33, 8.66)	.518	1.31 (0.21, 7.42)	.766	1.34 (0.22, 7.46)	.740	1.39 (1.07, 1.81)	. 015
No AST with no positive lymph nodes	Ref		Ref		Ref		Ref	
AST with no positive lymph nodes	2.74 (0.60, 14.48)	.198	2.07 (0.43, 10.89)	.363	2.71 (0.58, 14.46)	.206	0.95 (0.77, 1.17)	.618
No AST with positive lymph nodes	5.19 (0.85, 25.80)	.071	3.87 (0.64, 19.19)	.129	4.78 (0.77, 24.20)	.087	1.73 (0.88, 3.39)	.111
AST with positive lymph nodes	1.04 (0.24, 4.68)	.953	0.96 (0.22, 4.42)	.962	1.04 (0.24, 4.76)	.958	1.31 (1.09, 1.58)	.004
CPE	0.47 (0.23, 0.89)	.017			0.50 (0.24, 0.94)	.030		
PC1			1.40 (0.89, 2.16)	.143	1.26 (0.80, 1.94)	.310	1.09 (1.01, 1.18)	.020

Data are HR estimates with 95% CI between parentheses. CPE and PC1 are standardized, i.e. a one unit increase is equal to a one standard deviation increase in the HR estimate. Data are corrected for age, tumor size and grade, axillary load, surgery, radiotherapy and AST. HR = hazard ratio, OS = overall survival, Ref = reference, CI = confidence interval, AST = adjuvant systemic treatment, CPE = contralateral parenchymal enhancement, PC1 = principal component 1.

**Table 3:** Multivariable HR Estimates for IDFS and DRFS for CPE and PC1 (representative of proteasome gene expression) in three models of the MARGINS cohort with only CPE, only PC1, and both CPE and PC1.

	MARGINS (n = 221)						METABRIC (n = 1283)	
Variable	Model with CPE		Model with PC1		Model with CPE and PC1		Model with PC1	
	HR (95% CI)	P	HR (95% CI)	P	HR (95% CI)	P	HR (95% CI)	P
Age (years)	1.03 (0.99, 1.08)	.140	1.04 (1.00, 1.09)	.030	1.03 (0.99, 1.08)	.012	1.03 (1.02, 1.04)	<.001
Tumor size (mm)	1.03 (1.00, 1.05)	.070	1.03 (1.00, 1.05)	.033	1.02 (1.00, 1.05)	.184	1.02 (1.01, 1.02)	<.001
Tumor grade 1	Ref		Ref		Ref		Ref	
Tumor grade 2	1.48 (0.62, 3.73)	.377	1.65 (0.70, 4.23)	.270	1.61 (0.67, 4.11)	.858	1.21 (0.95, 1.54)	.127
Tumor grade 3	2.97 (0.88, 9.84)	.079	2.20 (0.58, 7.89)	.239	2.22 (0.59, 7.90)	.740	1.34 (1.04, 1.72)	.023
No AST with no positive lymph nodes	Ref		Ref		Ref		Ref	
AST with no positive lymph nodes	0.76 (0.22, 2.45)	.644	0.58 (0.16, 1.95)	.386	0.66 (0.18, 2.21)	.206	0.82 (0.67, 1.01)	.056
No AST with positive lymph nodes	2.93 (0.85, 8.60)	.084	2.45 (0.71, 7.18)	.144	2.59 (0.75, 7.63)	.087	1.20 (0.61, 2.36)	.587
AST with positive lymph nodes	0.55 (0.19, 1.55)	.261	0.51 (0.17, 1.47)	.216	0.54 (0.18, 1.54)	.958	1.16 (0.97, 1.39)	.098
CPE	0.69 (0.42, 1.06)	.097			0.78 (0.47, 1.21)	.030		
PC1			1.53 (1.08, 2.14)	.017	1.44 (1.07, 2.04)	.310	1.10 (1.02, 1.18)	.012

Data are HR estimates with 95% CI between parentheses. CPE and PC1 are standardized, i.e. a one unit increase is equal to a one standard deviation increase in the HR estimate. Data are corrected for age, tumor size and grade, axillary load, surgery, radiotherapy and AST. HR = hazard ratio, IDFS = invasive disease-free survival, Ref = reference, CI = confidence interval, AST = adjuvant systemic treatment, CPE = contralateral parenchymal enhancement, PC1 = principal component 1.

## 4 Discussion

CPE was most strongly associated with expression of the proteasome pathway in the tumor: high CPE (favorable prognosis) was associated with low proteasome gene expression in the MARGINS data. The association between tumor proteasome gene expression and survival was independently verified in the METABRIC data.

The proteasome is a protein complex that plays an essential role in the cellular protein homeostasis, regulating intracellular protein degradation, and is involved in processes such as apoptosis, cell cycle regulation, and angiogenesis [[37], [38], [39]]. Malignancies often exhibit increased proteasome activity to compensate for the aberrant protein synthesis and to maintain protein homeostasis [40]. Inhibition of the proteasome, e.g. through inhibition of nuclear factor-κB, will disrupt protein homeostasis and induce apoptosis in malignancies [39]. It has become a relatively novel target for cancer therapy [38,[41], [42], [43]]. Although proteasome inhibitors are currently approved for the treatment of multiple myeloma, and mantle-cell lymphoma, clinical efficacy with single-agent therapy is limited in solid tumors [39,44], including breast cancer [[45], [46], [47]]. Current efforts are aimed at combining proteasome inhibition with other therapeutic agents (i.e., endocrine therapy and chemotherapy) [44].

Increased proteasome activity is reported to be associated with poor prognosis in breast cancer [48,49]. Our results confirm these findings and suggest that CPE on MRI is associated with proteasome activity in the ER+/HER2-tumor. The proteasome plays an important role in the degradation and stability of the ER [50,51], and might play a role in acquired resistance against tamoxifen [52]. The role of the proteasome in ER turnover might explain why CPE was previously only associated with prognosis in ER+/HER2-breast cancer patients, although proteasome activity was also associated with prognosis in ER-breast cancer [19,48,49].

The proteasome pathway was previously associated with other features on MRI. Wu et al. observed that the proteasome pathway was significantly associated with imaging subtypes on breast MRI with distinct prognoses. These imaging subtypes were based on several quantitative imaging features, including ipsilateral parenchymal enhancement [19]. Quantitative analysis of the tumor and the ipsilateral parenchyma resulted in the identification of two imaging subtypes with minimal parenchymal enhancement and prominent parenchymal enhancement in which the proteasome pathway was significantly associated [19]. Our current work focused on one imaging feature (CPE) and its association with gene expression, future work will include multiple imaging features based on radiomics or other artificial intelligence (AI).

This study has several limitations. First, we have not validated the association between CPE and the proteasome gene expression pathway. Publicly available gene expression data matched with MRI data are limited. The Cancer Genome Atlas offers a public gene expression dataset matched with MRIs of The Cancer Imaging Archive, however, the number of available ER+/HER2-breast cancer patients withthe contralateral breast in the field of view is too small to achieve sufficient statistical power to validate the association. The current study should be considered hypothesis generating. Second, to facilitate the survival analysis the gene expression data was condensed into one PC to represent the pathway, which limits the interpretability and results in loss of information. Thirdly, patients received less endocrine therapy during the MARGINS study period compared to the study period of the METABRIC cohort. This may have influenced the survival analysis. Nonetheless, the association between CPE and survival was validated in an external cohort from the United States of America, in which a large number of patients received endocrine therapy (93%) [3]. Another limitation of this study was that we were unable to investigate whether CPE and proteasome gene expression are associated with (contralateral) breast cancer risk, because we did not have data on contralateral breast occurrence in the MARGINS cohort.

To conclude, high CPE on DCE-MRI was associated with low tumor proteasome gene expression pathways in unilateral ER+/HER2-breast cancer patients. Low proteasome gene expression in the tumor was associated with improved survival.

## Author contributions

Max A.A. Ragusi MD: Conceptualization, Methodology, Formal analysis, Investigation, Writing – original draft, Writing – review & editing. Tycho Bismeijer PhD: Conceptualization, Methodology, Formal analysis, Investigation, Data curation, Writing – review & editing. Bas H.M. van der Velden PhD: Conceptualization, Methodology, Formal analysis, Data curation, Writing – review & editing. Claudette E. Loo MD PhD: Investigation, Writing – review & editing, Resources. Sander Canisius PhD: Investigation, Writing – review & editing, Resources. Jelle Wesseling MD, PhD: Investigation, Writing – review & editing, Resources. Lodewyk F.A. Wessels PhD: Conceptualization, Methodology, Writing – review & editing, Supervision. Sjoerd G. Elias MD, PhD: Conceptualization, Methodology, Writing – review & editing, Supervision. Kenneth G.A. Gilhuijs PhD: Conceptualization, Methodology, Writing – review & editing, Supervision, Funding acquisition

## Funding

This work was supported by the Dutch Cancer Society (grant number 10755) and the 10.13039/501100003958Dutch Technology Foundation STW, which is part of the 10.13039/501100003246Netherlands Organisation for Scientific Research (10.13039/501100003246NWO), and partly funded by 10.13039/501100004725Ministry of Economic Affairs.

## Ethical approval

Informed consent was obtained and institutional review board approval was obtained.

## Declaration of competing interest

None declared.
