# Supplementary material for: Contralateral parenchymal enhancement on MRI is associated with tumor proteasome pathway gene expression and overall survival of early ER+/HER2-breast cancer patients
Source: Breast. 2021 Nov 2;60:230–7. doi: 10.1016/j.breast.2021.11.002 (PMC8591464; doi:10.1016/j.breast.2021.11.002)
Supplement: Multimedia component 1 [file mmc1.docx]

| **Supplemental Materials 1. Overview of all the genesets associated with CPE with an FDR < .25** | | | | | |
| --- | --- | --- | --- | --- | --- |
| **Geneset** | **Proteasome genes in the pathway** | **NES** | **Max ES at** | **LE** | **FDR** |
| KEGG_PROTEASOME | Yes | 2.04 | 4095 | .93 | .11 |
| REACTOME_GLUCONEOGENESIS | No | 1.98 | 2209 | .69 | .11 |
| REACTOME_AUF1_HNRNP_D0_BINDS_AND_DESTABILIZES_MRNA | Yes | 1.93 | 3877 | .77 | .11 |
| REACTOME_DEGRADATION_OF_AXIN | Yes | 1.93 | 3877 | .75 | .11 |
| REACTOME_VIF_MEDIATED_DEGRADATION_OF_APOBEC3G | Yes | 1.93 | 3877 | .77 | .11 |
| REACTOME_FBXL7_DOWN_REGULATES_AURKA_DURING_MITOTIC_ENTRY_AND_IN_EARLY_MITOSIS | Yes | 1.90 | 3877 | .77 | .11 |
| REACTOME_DEGRADATION_OF_GLI1_BY_THE_PROTEASOME | Yes | 1.90 | 4097 | .77 | .11 |
| REACTOME_DEFECTIVE_CFTR_CAUSES_CYSTIC_FIBROSIS | Yes | 1.89 | 4095 | .76 | .11 |
| REACTOME_REGULATION_OF_RUNX3_EXPRESSION_AND_ACTIVITY | Yes | 1.88 | 4095 | .75 | .11 |
| REACTOME_REGULATION_OF_RUNX2_EXPRESSION_AND_ACTIVITY | Yes | 1.87 | 4177 | .71 | .11 |
| REACTOME_CROSS_PRESENTATION_OF_SOLUBLE_EXOGENOUS_ANTIGENS_ENDOSOMES | Yes | 1.86 | 3877 | .79 | .11 |
| REACTOME_HEDGEHOG_LIGAND_BIOGENESIS | Yes | 1.85 | 3877 | .72 | .11 |
| REACTOME_REGULATION_OF_APOPTOSIS | Yes | 1.85 | 3877 | .75 | .11 |
| REACTOME_ACTIVATION_OF_NF_KAPPAB_IN_B_CELLS | Yes | 1.85 | 4127 | .74 | .11 |
| REACTOME_REGULATION_OF_PTEN_STABILITY_AND_ACTIVITY | Yes | 1.85 | 4177 | .72 | .11 |
| REACTOME_NEGATIVE_REGULATION_OF_NOTCH4_SIGNALING | Yes | 1.85 | 4095 | .74 | .11 |
| REACTOME_STABILIZATION_OF_P53 | Yes | 1.84 | 3877 | .71 | .11 |
| REACTOME_SCF_SKP2_MEDIATED_DEGRADATION_OF_P27_P21 | Yes | 1.83 | 3831 | .66 | .11 |
| REACTOME_DEGRADATION_OF_DVL | Yes | 1.82 | 4095 | .73 | .11 |
| REACTOME_DECTIN_1_MEDIATED_NONCANONICAL_NF_KB_SIGNALING | Yes | 1.82 | 4127 | .73 | .11 |
| REACTOME_METABOLISM_OF_POLYAMINES | Yes | 1.82 | 4109 | .72 | .11 |
| REACTOME_REGULATION_OF_RAS_BY_GAPS | Yes | 1.80 | 4095 | .70 | .12 |
| REACTOME_ABC_TRANSPORTER_DISORDERS | Yes | 1.80 | 4095 | .66 | .12 |
| REACTOME_THE_ROLE_OF_GTSE1_IN_G2_M_PROGRESSION_AFTER_G2_CHECKPOINT | Yes | 1.80 | 3877 | .69 | .12 |
| REACTOME_APC_C:CDH1_MEDIATED_DEGRADATION_OF_CDC20_AND_OTHER_APC_C:CDH1_TARGETED_PROTEINS_IN_LATE_MITOSIS_EARLY_G1 | Yes | 1.79 | 3877 | .66 | .12 |
| REACTOME_ANTIGEN_PROCESSING_CROSS_PRESENTATION | Yes | 1.79 | 3910 | .66 | .12 |
| REACTOME_ASYMMETRIC_LOCALIZATION_OF_PCP_PROTEINS | Yes | 1.79 | 4095 | .71 | .12 |
| REACTOME_CELLULAR_RESPONSE_TO_HYPOXIA | Yes | 1.78 | 3877 | .66 | .12 |
| REACTOME_CDK_MEDIATED_PHOSPHORYLATION_AND_REMOVAL_OF_CDC6 | Yes | 1.76 | 3877 | .64 | .13 |
| REACTOME_ENDOSOMAL_VACUOLAR_PATHWAY | No | 1.75 | 3162 | .91 | .13 |
| REACTOME_DOWNSTREAM_SIGNALING_EVENTS_OF_B_CELL_RECEPTOR_BCR | Yes | 1.75 | 4127 | .66 | .13 |
| REACTOME_ORC1_REMOVAL_FROM_CHROMATIN | Yes | 1.73 | 3877 | .60 | .15 |
| REACTOME_G1_S_DNA_DAMAGE_CHECKPOINTS | Yes | 1.73 | 3877 | .63 | .15 |
| REACTOME_RUNX1_REGULATES_TRANSCRIPTION_OF_GENES_INVOLVED_IN_DIFFERENTIATION_OF_HSCS | Yes | 1.72 | 4327 | .68 | .15 |
| REACTOME_ASSEMBLY_OF_THE_PRE_REPLICATIVE_COMPLEX | Yes | 1.72 | 3877 | .61 | .15 |
| BIOCARTA_TGFB_PATHWAY | No | 1.71 | 2544 | .63 | .15 |
| REACTOME_SIGNALING_BY_THE_B_CELL_RECEPTOR_BCR | Yes | 1.71 | 4127 | .60 | .15 |
| REACTOME_ACTIVATION_OF_APC_C_AND_APC_C:CDC20_MEDIATED_DEGRADATION_OF_MITOTIC_PROTEINS | Yes | 1.71 | 3877 | .62 | .15 |
| REACTOME_HEDGEHOG_OFF_STATE | Yes | 1.71 | 4097 | .62 | .15 |
| REACTOME_CYTOSOLIC_TRNA_AMINOACYLATION | No | 1.70 | 3736 | .63 | .15 |
| REACTOME_HEDGEHOG_ON_STATE | Yes | 1.70 | 4588 | .66 | .15 |
| REACTOME_REGULATION_OF_PYRUVATE_DEHYDROGENASE_PDH_COMPLEX | No | 1.70 | 2742 | .53 | .15 |
| REACTOME_ANTIGEN_PRESENTATION:_FOLDING_ASSEMBLY_AND_PEPTIDE_LOADING_OF_CLASS_I_MHC | No | 1.69 | 3108 | .64 | .15 |
| REACTOME_DEGRADATION_OF_BETA_CATENIN_BY_THE_DESTRUCTION_COMPLEX | Yes | 1.69 | 3877 | .60 | .15 |
| REACTOME_FCERI_MEDIATED_NF_KB_ACTIVATION | Yes | 1.68 | 4127 | .62 | .16 |
| REACTOME_INTERLEUKIN_1_SIGNALING | Yes | 1.67 | 4222 | .61 | .17 |
| REACTOME_DEGRADATION_OF_CYSTEINE_AND_HOMOCYSTEINE | No | 1.67 | 2271 | .46 | .17 |
| REACTOME_SWITCHING_OF_ORIGINS_TO_A_POST_REPLICATIVE_STATE | Yes | 1.65 | 3877 | .54 | .20 |
| REACTOME_TRANSCRIPTIONAL_REGULATION_BY_RUNX2 | Yes | 1.64 | 4177 | .55 | .20 |
| REACTOME_REGULATION_OF_MITOTIC_CELL_CYCLE | Yes | 1.64 | 3877 | .58 | .20 |
| REACTOME_UCH_PROTEINASES | Yes | 1.63 | 4366 | .62 | .21 |
| REACTOME_MAPK3_ERK1_ACTIVATION | No | 1.63 | 855 | .50 | .21 |
| REACTOME_FORMATION_OF_ATP_BY_CHEMIOSMOTIC_COUPLING | No | 1.63 | 3755 | .72 | .21 |
| REACTOME_SIGNALING_BY_NOTCH4 | Yes | 1.62 | 3091 | .48 | .22 |
| REACTOME_PYRUVATE_METABOLISM | No | 1.61 | 2977 | .54 | .23 |
| REACTOME_PCP_CE_PATHWAY | Yes | 1.61 | 3947 | .59 | .23 |
| BIOCARTA_CCR3_PATHWAY | No | 1.61 | 3675 | .63 | .24 |
| REACTOME_ABC_FAMILY_PROTEINS_MEDIATED_TRANSPORT | Yes | 1.60 | 3930 | .55 | .24 |
| REACTOME_ENDOSOMAL_SORTING_COMPLEX_REQUIRED_FOR_TRANSPORT_ESCRT | No | 1.60 | 2341 | .42 | .24 |
| REACTOME_CLEC7A_DECTIN_1_SIGNALING | Yes | 1.60 | 4127 | .55 | .24 |
| REACTOME_REGULATION_OF_MRNA_STABILITY_BY_PROTEINS_THAT_BIND_AU_RICH_ELEMENTS | Yes | 1.59 | 3877 | .54 | .24 |
| REACTOME_STING_MEDIATED_INDUCTION_OF_HOST_IMMUNE_RESPONSES | No | 1.59 | 408 | .20 | .24 |
| REACTOME_TRANSCRIPTIONAL_REGULATION_BY_RUNX3 | Yes | 1.59 | 4227 | .54 | .24 |
| REACTOME_TIGHT_JUNCTION_INTERACTIONS | No | 1.59 | 2983 | .58 | .24 |
| REACTOME_DNA_REPLICATION_PRE_INITIATION | Yes | 1.59 | 3877 | .52 | .24 |
| REACTOME_CYCLIN_A:CDK2_ASSOCIATED_EVENTS_AT_S_PHASE_ENTRY | Yes | 1.58 | 3831 | .54 | .25 |
| REACTOME_TNFR2_NON_CANONICAL_NF_KB_PATHWAY | Yes | 1.58 | 4171 | .54 | .25 |
| BIOCARTA_IL3_PATHWAY | No | 1.58 | 4836 | .85 | .25 |
| REACTOME_NOTCH2_INTRACELLULAR_DOMAIN_REGULATES_TRANSCRIPTION | No | 1.58 | 1460 | .40 | .25 |
| REACTOME_SIGNALING_BY_HEDGEHOG | Yes | 1.57 | 3877 | .53 | .25 |
| REACTOME_TRANSCRIPTIONAL_REGULATION_BY_RUNX1 | Yes | 1.57 | 4095 | .52 | .25 |
| REACTOME_MAPK6_MAPK4_SIGNALING | Yes | 1.57 | 4227 | .60 | .25 |
| REACTOME_PTEN_REGULATION | Yes | 1.57 | 4177 | .53 | .25 |
| REACTOME_JOSEPHIN_DOMAIN_DUBS | No | 1.57 | 2936 | .64 | .25 |
| REACTOME_DOWNSTREAM_TCR_SIGNALING | Yes | 1.57 | 4127 | .56 | .25 |
| BIOCARTA_EGFR_SMRTE_PATHWAY | No | 1.56 | 1950 | .55 | .25 |
| REACTOME_INTERLEUKIN_12_SIGNALING | Yes | 1.56 | 1803 | .38 | .25 |
| REACTOME_REGULATION_OF_RUNX1_EXPRESSION_AND_ACTIVITY | No | 1.56 | 3297 | .53 | .25 |
| CPE = contralateral parenchymal enhancement, FDR = false discovery rate, NES = normalized enrichment statistic, Max ES at = maximum enrichment score at, LE = leading edge. | | | | | |
