# Supplementary material for: Contralateral parenchymal enhancement on MRI is associated with tumor proteasome pathway gene expression and overall survival of early ER+/HER2-breast cancer patients
Source: Breast. 2021 Nov 2;60:230–7. doi: 10.1016/j.breast.2021.11.002 (PMC8591464; doi:10.1016/j.breast.2021.11.002)
Supplement: Multimedia component 2 [file mmc2.docx]

| **Supplemental Materials 2. Overview of the Pearson’s correlation of CPE with all genes in the KEGG_PROTEASOME pathway** | | | |
| --- | --- | --- | --- |
| **Gene** | **Correlation** | **95% Confidence interval** | **p** |
| ENSG00000205220 | -.389 | -.495, -.273 | < .001 |
| ENSG00000129084 | -.329 | -.440, -.207 | < .001 |
| ENSG00000142507 | -.282 | -.398, -.158 | < .001 |
| ENSG00000240065 | -.277 | -.393, -.152 | < .001 |
| ENSG00000163636 | -.265 | -.382, -.139 | < .001 |
| ENSG00000204264 | -.261 | -.378, -.135 | < .001 |
| ENSG00000100567 | -.258 | -.375, -.131 | < .001 |
| ENSG00000277791 | -.245 | -.364, -.118 | < .001 |
| ENSG00000103035 | -.219 | -.34. -.091 | .001 |
| ENSG00000165916 | -.216 | -.337, -.088 | .001 |
| ENSG00000132963 | -.211 | -.332, -.083 | .001 |
| ENSG00000100804 | -.203 | -.325, -.074 | .002 |
| ENSG00000013275 | -.194 | -.316, -.065 | .003 |
| ENSG00000115233 | -.179 | -.302, -.049 | .007 |
| ENSG00000127922 | -.162 | -.287, -.032 | .015 |
| ENSG00000100764 | -.149 | -.275, -.019 | .025 |
| ENSG00000136930 | -.141 | -.267, -.011 | .033 |
| ENSG00000087191 | -.130 | -.256, .001 | .052 |
| ENSG00000008018 | -.123 | -.250, .007 | .065 |
| ENSG00000108671 | -.115 | -.242, .016 | .085 |
| ENSG00000185627 | -.115 | -.241, .016 | .086 |
| ENSG00000197170 | -.110 | -.237, .021 | .100 |
| ENSG00000143106 | -.110 | -.237, .021 | .100 |
| ENSG00000100911 | -.106 | -.233, .025 | .112 |
| ENSG00000126067 | -.105 | -.233, .025 | .114 |
| ENSG00000101182 | -.105 | -.232, .026 | .116 |
| ENSG00000108344 | -.100 | -.228, .031 | .133 |
| ENSG00000100902 | -.100 | -.228, .031 | .133 |
| ENSG00000161057 | -.099 | -.226, .032 | .140 |
| ENSG00000131467 | -.090 | -.218, .041 | .176 |
| ENSG00000041357 | -.084 | -.212, .047 | .207 |
| ENSG00000173692 | -.076 | -.205, .055 | .253 |
| ENSG00000159352 | -.070 | -.199, .061 | .294 |
| ENSG00000099341 | -.069 | -.198, .062 | .299 |
| ENSG00000125818 | -.068 | -.196, .063 | .311 |
| ENSG00000106588 | -.055 | -.184, .076 | .410 |
| ENSG00000068878 | -.050 | -.180, .081 | .452 |
| ENSG00000092010 | -.047 | -.177, .084 | .478 |
| ENSG00000159377 | -.034 | -.164, .097 | .611 |
| ENSG00000175166 | -.031 | -.161, .100 | .644 |
| ENSG00000100519 | .009 | -.122, .139 | .897 |
| CPE = contralateral parenchymal enhancement. | | | |
